# Understanding the Role of Parent‒Child Relationships in Conscientiousness and Neuroticism Development among Chinese Middle School Students: A Cross-Lagged Model

**DOI:** 10.3390/bs13100876

**Published:** 2023-10-23

**Authors:** Xiaojie Cao, Xinqiao Liu

**Affiliations:** 1 Graduate School of Education, Peking University, Beijing 100871, China; 2 School of Education, Tianjin University, Tianjin 300350, China

**Keywords:** parent‒child relationships, conscientiousness, neuroticism, students

## Abstract

The parent‒child relationship is a crucial factor in promoting adolescent mental health. However, the current evidence on the relationship between parent‒child relationships and adolescent conscientiousness and neuroticism, as well as the directionality of these relationships, remains limited. In particular, there is a lack of analysis focusing on Chinese middle school students. Based on a sample of 8437 students from the China Education Panel Survey (CEPS) database, this study empirically examined the bidirectional relationships between parent‒child relationships, conscientiousness and neuroticism among Chinese middle school students, with specific emphasis on the significant role of parent‒child relationships in the development of conscientiousness and neuroticism. Descriptive statistical results indicated that during the seventh and eighth grades of Chinese middle school students, the closeness of their parent‒child relationships with both parents decreased, while the level of conscientiousness showed a slight decrease, and neuroticism showed an increasing trend. Correlational results demonstrated a significant positive correlation between parent‒child relationships and conscientiousness and a significant negative correlation between parent‒child relationships and neuroticism. Further analysis using cross-lagged models revealed that parent‒child relationships significantly positively predicted subsequent conscientiousness development, and conscientiousness significantly positively predicted subsequent parent‒child relationships. Parent‒child relationships significantly negatively predicted subsequent neuroticism development, and neuroticism levels also significantly negatively predicted subsequent parent‒child relationships. Based on these findings, we believe that there is a need to strengthen parent‒child relationships and to recognize the important role that both mothers and fathers play in the healthy development of their children. Both parents should actively contribute to their children’s upbringing and take responsibility for their family education.

## 1. Introduction

Currently, the rising prevalence of mental health problems among adolescents worldwide is a growing concern and indicates a trend toward younger age cohorts [1,2,3,4]. According to statistics released by the World Health Organization, approximately one in every seven adolescents between the ages of 10 and 19 suffers from mental health problems [5]. A recent meta-analysis study of 96,000 adolescents from 11 countries found that almost 90% of the 21 studies covering 23 indicators of anxiety, depression and general mental health exhibited an upward trend. This suggests a significant escalation in the severity of negative emotions and mental health problems among adolescents [6]. Specifically, a national social survey conducted in Iceland found that between 2016 and 2020, the proportion of 13-year-old Icelandic adolescents with depression symptoms increased from 16.52% to 18.72%, while mental well-being decreased from 25.21% to 23.13% [1]. Some studies have also highlighted the exacerbation of adolescent mental health problems due to prolonged home confinement and deteriorating family dynamics during the COVID-19 pandemic [7]. For instance, the prevalence of depression and anxiety symptoms in Chinese adolescents increased significantly from 36.6% to 57.0% and from 19% to 36.7%, respectively, leading to a significant increase in the risk of emotional dysregulation [8].

Conscientiousness and neuroticism, the integral parts of the widely recognized Big Five personality model, play contrasting roles in adolescent mental health [9,10,11]. Conscientiousness typically refers to an individual’s tendencies toward diligence, self-discipline, caution and self-control, while neuroticism manifests as emotional traits, such as anxiety, depression, hostility, inhibition and vulnerability [12,13,14,15]. Specifically, conscientiousness serves as a protective factor in mitigating the impact of negative emotions on adolescents. Adolescents with high levels of conscientiousness are more likely to exhibit positive psychological traits, such as emotional stability, self-management skills and effective coping strategies [11,16,17,18]. Conversely, neuroticism has long-term detrimental effects on individual development, including reduced quality of life, poorer health, increased frequency of experiencing unpleasant events and a potentially shortened life span. Individuals with higher levels of neuroticism are more likely to experience negative emotions, show symptoms of depression and anxiety, and suffer from mental health problems [19,20,21,22,23]. Therefore, there is an urgent need to identify the key factors for preventing the exacerbation of neuroticism in adolescents and fostering conscientiousness development to effectively reduce the occurrence of negative emotions, promote adolescent mental health and facilitate holistic development.

It is worth noting that adolescence is a critical period for individual development and personality formation [3,4,11,24], where the parent‒child relationship is considered a key factor, which may influence adolescent development and mental health [25,26,27,28]. The parent‒child relationship encompasses the emotional, communicative and interactive aspects between children and their parents. The ecological systems theory posits that parents are not only the earliest socializing agents for individuals but also play a crucial role in the microsystem of the family [29]. In China in particular, early attachment formation is influenced by long-standing family-oriented values. The impact of the parent‒child relationships formed by the emphasis on shared parenting in everyday life cannot be overlooked in individual development. According to the stress-buffering model, social support or positive relationships with others can mitigate the potentially detrimental impacts of stressful events [30]. In the context of the family environment, the support and warmth derived from close parent‒child relationships may help alleviate the stress, which adolescents encounter in school or peer relationships. However, further empirical research is necessary to validate these assumptions. Previous research has focused on the influence of parent‒child relationships on adolescent aggression, maladaptive behaviors, social–emotional difficulties and general levels of mental health [31,32,33,34], but specific investigations of their effects on the development of conscientiousness and neuroticism are lacking. In reality, early adolescence marks a critical juncture characterized by physiological changes, academic adjustment and peer pressure, leading to crises and an urgent need for attention in parent‒child relationships [9]. On the one hand, as individuals in adolescence increasingly yearn for independence and seek autonomy, attachment to parents diminishes with age, resulting in more arguments and fewer opportunities for parent‒child communication [16,17,33,35]. On the other hand, in countries such as China, with limited educational resources and intense competition, parents have high expectations for their children’s education and often demand outstanding academic performance, which puts enormous pressure on children [36,37] and hinders the establishment of harmonious parent‒child relationships. Similarly, in the case of Bangladeshi middle school students, a mixed-methods study reveals that parents willingly bear significant financial burdens to enroll their children in extracurricular tutoring in order to enhance academic competitiveness. This places considerable demand on the students, who have to cope with a heavy academic workload even in their leisure time, sacrificing adequate rest and leading to profound psychological stress [38]. A study of Canadian adolescents further found that parent–child relationships tend to become rigid, especially when parents impose unrealistic educational expectations, increasing students’ vulnerability to self-injury behaviors [39]. The evidence from German fifth graders suggests that positive parent–child relationships and parental understanding of real needs are essential prerequisites for promoting children’s psychological well-being [40]. Therefore, it is important to understand the current status of parent‒child relationships among middle school students and their role in the development of conscientiousness and neuroticism.

Furthermore, previous literature has often focused broadly on parent–child relationships without distinguishing between maternal and paternal roles. The present study addresses the growing need for a more specific understanding of the effect of mother–child and father–child relationships on adolescent development. In China, mothers are typically seen as the primary caregivers and nurturers, while fathers often take on more peripheral roles [41]. There is an increasing interest in elucidating the precise contributions of both parents to adolescent development. Our study refines the investigation of the role of parent–child relationships in conscientiousness and neuroticism development by distinguishing between mother–child and father–child relationships separately. This is also in line with the policy direction of the Chinese government, which emphasizes the collective involvement of parents in fostering the development of minors.

## 2. Literature Review

### 2.1. Parent‒Child Relationship and Neuroticism

Previous literature has extensively discussed the influence of parent‒child relationships on adolescent neuroticism, but there is little evidence on the influence of neuroticism on parent‒child relationships. In an autobiographical review of the risk factors for depression, Hammen (2018) suggests that experiences of rejection, exclusion and conflict in interpersonal relationships, particularly parent‒child relationships, can shape negative cognitive patterns and self-evaluations, leading to higher levels of depression risk [42]. Warm parent‒child relationships can act as a protective factor against the negative effects of stress [43]. Meanwhile, some empirical studies have focused on the relationship between parent‒child relationships and adolescent mental health, particularly negative emotional traits, but most of them have found only correlational relationships. For instance, Li et al. (2020) used cross-sectional data to show a positive correlation between improved parent‒child relationships and better mental health among Chinese middle school students [33]. A study involving 234 British adolescents aged approximately 18 found a significant negative correlation between parent‒child relationships and adolescent neuroticism [44]. Another study of public high school students in New York revealed that a lack of intimacy with parents was associated with a higher degree of depressive mood [45]. The evidence from a study of 847 Israeli adolescents showed that adolescents with more intimate parental relationships experienced less distress, higher levels of happiness and more social support [28]. In addition, previous literature has analyzed the unidirectional predictive relationship between parent‒child relationships and neuroticism. For example, a longitudinal study of nearly 700 adolescents from New Jersey and Colorado found that high levels of parent‒child bonding (i.e., closer parent‒child relationships) could protect adolescents from the negative effects of peer stressors and reduce depressive symptoms [9]. A survey of 290 European–American adolescents found that warm parental behaviors significantly predicted an increase in adolescent optimism and a decrease in neuroticism levels [25]. A one-year longitudinal study involving a sample of 418 participants found that the quality of parent‒child relationships in eighth grade significantly predicted depressive mood in ninth grade [46]. However, these studies have limitations, such as insufficient representativeness of the sample and lack of generalizability of the conclusions [44]. As mentioned earlier, given the importance of measuring the quality of parent‒child relationships, it is necessary to examine the bidirectional relationship between the closeness level of parent‒child relationships and adolescent neuroticism. Unfortunately, previous research has rarely investigated the dynamic relationship between these two variables over time, and in particular, empirical evidence on the influence of neuroticism on parent‒child relationships is lacking [16]. This is the gap, which the current study aims to address and analyze.

### 2.2. Parent‒Child Relationships and Conscientiousness

There is currently limited direct research on the bidirectional relationship between parent‒child relationships and conscientiousness development, although most of the literature suggests a potential positive correlation between these two variables. For example, a survey of nearly 1000 Chinese primary school students confirmed the role of family environmental factors in shaping conscientiousness, revealing a significant positive correlation between parent‒child relationships and children’s levels of conscientiousness [47]. Another study of some 750 people in the community also found a significant positive correlation between parent‒child relationships and conscientiousness, with respondents reporting lower scores in conscientiousness among those who reported lower levels of parental care and higher levels of parental intrusiveness [48]. As an essential component of the family ecological environment, the parent‒child relationship has a positive impact on individual adjustment. Compared with unhealthy and negative parent‒child relationships, close and positive parent‒child relationships can facilitate the development of individual adaptive abilities [9]. Additionally, some studies have examined the association between parental nurturing behaviors, family environmental characteristics and adolescent conscientiousness, reflecting to some extent how positive parent‒child relationships contribute to the development of adolescent conscientiousness. A survey conducted among 674 participants aged 18 to 28 from three universities in Slovenia revealed a negative correlation between conscientiousness and perceived parental intrusiveness and fear of disappointment from mothers, suggesting that individuals with lower levels of conscientiousness may have poorer relationships with their parents [16]. Studies conducted in Serbia and Germany found a positive correlation between adult conscientiousness and a supportive family environment in childhood [18]. A survey of 402 adolescents aged 14 to 21 found significant positive correlations between conscientiousness (including resilience) and both mother support and father support [49]. Although a study of 287 Dutch families also found an association between the quality of the parent‒child relationship and conscientiousness, the association was relatively weak [50]. Furthermore, a meta-analysis conducted by Prinzie et al. (2009) found that higher levels of conscientiousness and lower levels of neuroticism in parents were associated with greater warmth and behavioral control, while lower levels of neuroticism were associated with greater autonomy support. However, it is important to note that this study focused on parents as subjects, whereas in reality, as the recipients of education, children’s personality traits and their development also deserve attention [27]. In summary, there is currently a lack of literature based on longitudinal data, which reveals the bidirectional relationship between parent‒child relationships and conscientiousness, and empirical analysis is needed to provide new evidence. The existing research mainly focuses on Western countries and includes a wide age range, making it difficult to directly apply the conclusions to the adolescent population in the Chinese context. This highlights the need for further exploration and investigation in this study.

### 2.3. Research Hypotheses

Our current understanding of the relationship between parent‒child relationships and adolescent neuroticism and conscientiousness is still quite limited. In particular, there is a lack of empirical evidence regarding the bidirectional relationships among these three variables in the context of Chinese education. It is imperative to gain a deeper understanding of the relationships between parent‒child relationships, conscientiousness and neuroticism among Chinese middle school students, especially in the approximate seventh grade. Adolescents in this age group are in a critical period of psychological well-being, academic adjustment and personality development [3,11,51]. Previous literature has shown that the transition from childhood to adolescence is accompanied by changes in personality traits [52]. Investigations involving German and Spanish students have indicated a slight decrease in conscientiousness levels between the ages of 12 and 15 [53], while neuroticism, which is characterized by anxiety, emotional instability and vulnerability, is increasing worldwide [54]. At the same time, early adolescence is often a high-risk period for a decline in the intimacy of parent–child relationships and the emergence of crises [16,17,33,35]. An analysis based on changes in parent–child relationships among over 1000 American adolescents found a downward trend in the level of closeness in parent–child relationships from early childhood to age 15 [55]. Since the parent‒child bond is the closest and undoubtedly the longest lasting interpersonal relationship for adolescents, we speculate that the parent–child relationship may play a significant role in the development of adolescent conscientiousness and neuroticism based on the above literature review and theory. The establishment of close parent–child relationships within the family context may serve as a protective buffer against negative emotions experienced by adolescents in educational and other environments. Consequently, improving parent–child relationships and implementing effective family education strategies hold the potential to guide children along a positive trajectory of personal growth. Additionally, if decreasing conscientiousness and increasing neuroticism serve as indicators of potential problems within the parent–child relationship, this offers precise guidance for timely intervention and improvement through family education.

In conclusion, the present study aims to systematically explore the bidirectional relationships between parent–child relationships, neuroticism and conscientiousness, with a particular focus on elucidating the role of parent–child relationships in the development of these traits during adolescence. To address these key questions, we use a cross-lagged model to clarify the bidirectional predictive relationships between parent‒child relationships and the development of conscientiousness and neuroticism among Chinese middle school students. The findings provide both theoretical and practical implications for future research efforts. Based on the literature and research objectives, we propose the following research hypotheses:

**Hypothesis** **1.** *During the seventh and eighth grades, there is a slight decrease in the level of closeness in parent‒child relationships, a decrease in conscientiousness and an increase in neuroticism among Chinese middle school students.*

**Hypothesis** **2.** *There is a significant positive correlation between parent‒child relationships and conscientiousness and a significant negative correlation between parent‒child relationships and neuroticism among Chinese middle school students.*

**Hypothesis** **3.** *There is a bidirectional relationship between parent‒child relationships and both conscientiousness and neuroticism development among Chinese middle school students.*

**Hypothesis** **3a.** *The closeness of parent‒child relationships in grade seven can significantly positively predict the level of conscientiousness in grade eight among middle school students, and the level of conscientiousness in grade seven can significantly positively predict the closeness of parent‒child relationships in grade eight.*

**Hypothesis** **3b.** *The closeness of parent‒child relationships in grade seven can significantly negatively predict the level of neuroticism in grade eight among middle school students, and the level of neuroticism in grade seven can significantly negatively predict the closeness of parent‒child relationships in grade eight.*

## 3. Methods

### 3.1. Participants

The dataset used in this study is the China Education Panel Survey (CEPS) database. CEPS is a nationally representative longitudinal survey project, which has gained widespread recognition. It was designed by the National Survey Research Center (NSRC) of the Renmin University of China. The project is currently ongoing and has released data for two waves, namely the 2013–2014 and 2014–2015 periods. Taking the 2013–2014 academic year as the baseline, CEPS selected two concurrent cohorts of students, namely grade seven and grade nine in middle school, as the starting point of the survey. The sampling procedure used a multistage probability proportional to size and whole-class sampling method, with average educational attainment and the proportion of floating population as stratification variables. A follow-up survey was then conducted in 2014–2015. It is important to note that the follow-up survey followed only those students who were in grade seven at baseline. The CEPS database provides the necessary data support to investigate the quality of parent‒child relationships and the psychological well-being of Chinese middle school students in this study. We selected students who participated in both waves of the survey (i.e., grade seven at baseline and grade eight at the follow-up survey), and we also excluded samples with missing variables. The final sample consisted of 8437 students.

### 3.2. Measures

#### 3.2.1. Parent‒Child Relationship

The CEPS student questionnaire contains separate questions on the parent‒child relationship with the mother and the father. Specifically, we used the following questions from the questionnaire: “How is the general relationship between you and your parents (Relationship between you and your mother)?” and “How is the general relationship between you and your parents (Relationship between you and your father)?”. Each question had three response options: “1 = Not close”, “2 = Not too close nor too far” and “3 = Very close”. Higher scores indicate a closer relationship between the child and the parent, indicating a better parent‒child relationship.

#### 3.2.2. Neuroticism

To measure the levels of neuroticism, we used the following questions from the CEPS student questionnaire: “Did you feel blue in the past seven days?”; “Did you feel unhappy in the past seven days?”; “Did you feel depressed in the past seven days?”; “Did you feel that life was meaningless in the past seven days?”; “Did you feel sad in the past seven days?”; and “I feel bored in this school”. The response options for the first five questions were on a 5-point scale ranging from “1 = never” to “5 = always”. The last question had a 4-point scale with the following response options: “1 = Strongly disagree”, “2 = Somewhat disagree”, “3 = Somewhat agree” and “4 = Strongly agree”. The average score across these six questions was calculated, with higher values indicating a higher level of neuroticism. The reliability coefficients α for neuroticism in the first and second years were 0.827 and 0.876, respectively, indicating a high level of measurement reliability.

#### 3.2.3. Conscientiousness

To measure conscientiousness, we used the following questions from the CEPS student questionnaire: “I would try my best to go to school even if I was not feeling very well or had other reasons to stay at home”; “I would try my best to finish even the homework I dislike”; and “I would try my best to finish my homework, even if it would take me quite a long time”. Each question had a 4-point scale ranging from “1 = Strongly disagree” to “4 = Strongly agree”. The average score across these questions was calculated, with higher scores indicating higher levels of conscientiousness. The reliability coefficients α for conscientiousness in the first and second years were 0.690 and 0.810, respectively, indicating good measurement reliability.

### 3.3. Data Analysis

This study used Stata 17.0 and Mplus 7.4 software for data analysis. The dataset used was sourced from the CEPS database, which provided the secondary data for our analysis. We first conducted a descriptive analysis of parent‒child relationships, neuroticism and conscientiousness among Chinese middle school students in grades seven and eight to gain a basic understanding of these variables. Building upon this foundation, we conducted a correlation analysis to examine the associations between parent‒child relationships, neuroticism and conscientiousness. Finally, in line with the research objectives and hypotheses, we used a cross-lagged model to analyze the bidirectional predictive relationships among parent‒child relationships, neuroticism and conscientiousness, with the aim of elucidating the role of parent‒child relationships in the development of conscientiousness and neuroticism among Chinese middle school students. This study obtained ethical approval from the local Institutional Review Board (IRB).

### 3.4. Cross-Lagged Model

The cross-lagged model is a longitudinal research model, which examines the existence and direction of the relationship between variables. In the context of this model, if X and Y represent two variables, and T1 and T2 represent two points in time (T1 is earlier than T2), the cross-lagged model essentially compares the relationship between X at time T1 and Y at time T2 and the relationship between Y at time T1 and X at time T2, thus helping clarify how X and Y interact with each other. The present study focuses on the variables of parent‒child relationships, neuroticism and conscientiousness; therefore, we use the cross-lagged model to investigate the predictive effects of parent‒child relationships on neuroticism and conscientiousness and to clarify the bidirectional relationship between these variables among Chinese middle school students.

## 4. Results

### 4.1. Descriptive Statistics of Parent‒Child Relationships, Neuroticism and Conscientiousness

Table 1 presents descriptive statistics, including the mean scores, standard deviations and other indicators, for parent‒child relationships, neuroticism and conscientiousness among Chinese middle school students. The mean scores for parent‒child relationships with mothers in grades seven and eight were 2.747 and 2.707, respectively, while the mean scores for parent‒child relationships with fathers in grades seven and eight were 2.629 and 2.506, respectively. It can be observed that the level of closeness in parent‒child relationships is moderate, with slightly higher levels of closeness reported between middle school students and their mothers compared to their fathers. In addition, there is a slight decrease in the level of closeness in parent‒child relationships as the school level increases. Meanwhile, the conscientiousness of middle school students shows a decline between grades seven and eight, with the average score dropping from 3.424 to 3.155. It is worth noting that there is a slight upward trend in neuroticism levels among middle school students, as the average score increases from 1.920 in grade seven to 2.095 in grade eight. Additionally, according to independent sample t-tests, there were significant differences in parent–child relationship (mother) (t = 5.303, *p* = 0.000), parent–child relationship (father) (t = 14.050, *p* = 0.000), conscientiousness (t = 25.373, *p* = 0.000) and neuroticism (t = −14.809, *p* = 0.000) between the first and second wave. This trend may reflect the changes in family interactions and psychological development, which middle school students experience at this stage, highlighting the need for timely attention from parents and teachers. Hypothesis 1 was confirmed.

### 4.2. Correlation Analysis of Parent‒Child Relationships, Neuroticism and Conscientiousness

Table 2 shows the correlation coefficients between parent–child relationships, neuroticism and conscientiousness among Chinese middle school students. According to Cohen’s guidelines, correlation coefficients of 0.1, 0.3 and 0.5 are considered small, medium and large effect sizes, respectively [56]. The data demonstrate significant negative correlations (*p* < 0.01) between parent–child relationship (mother) and neuroticism, as well as significant positive correlations (*p* < 0.01) between parent–child relationship (mother) and conscientiousness each year. Similar patterns are observed for the parent–child relationship (father). There is also a significant negative correlation (*p* < 0.01) between conscientiousness and neuroticism. In the first year, the effect sizes between parent–child relationship (mother) and conscientiousness (r = 0.105), neuroticism (r = −0.241), as well as between parent–child relationship (father) and conscientiousness (r = 0.109), neuroticism (r = −0.249), are relatively small. The effect size between conscientiousness and neuroticism is also relatively small (r = −0.169). In the second year, similar trends of small effect sizes are observed. Additionally, both parent‒child relationships with mothers and fathers in the first year are significantly positively correlated with conscientiousness in the second year and significantly negatively correlated with neuroticism in the second year (*p* < 0.01). The correlations of parent–child relationship (mother) in the first year with conscientiousness and neuroticism in the second year are 0.100 and −0.157, respectively. The correlations of parent–child relationship (father) in the first year with conscientiousness and neuroticism in the second year are 0.104 and −0.163, respectively, falling within the range of small effect sizes. In summary, it is evident that the negative correlation between parent‒child relationships and neuroticism, as well as the positive correlation between parent‒child relationships and conscientiousness, remain stable among Chinese middle school students in grades seven and eight. Moreover, there are significant correlations (*p* < 0.01) between these three variables across the years. Hypothesis 2 was also confirmed.

### 4.3. Cross-Lagged Panel Model of Parent‒Child Relationships, Neuroticism and Conscientiousness

**Figure 1:**
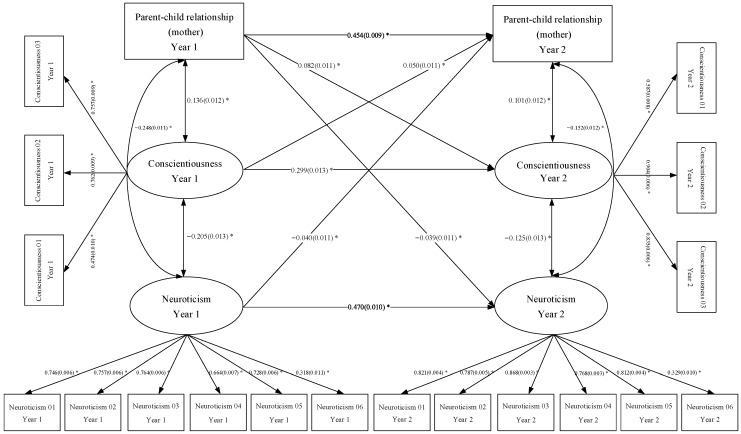
Bidirectional relationships between the parent‒child relationship (mother), neuroticism and conscientiousness. Note: (1) The variables Conscientiousness 01, Conscientiousness 02 and Conscientiousness 03 in the figure represent the questionnaire items used to measure conscientiousness; the variables Neuroticism 01 to Neuroticism 06 in the figure represent the questionnaire items used to measure neuroticism. (2) The numbers in brackets in the figure represent Standard Error (S.E.). (3) We use “*” to indicate a statistically significant relationship (*p* < 0.05) between variables. Figure 2 is the same as Figure 1.

**Figure 2:**
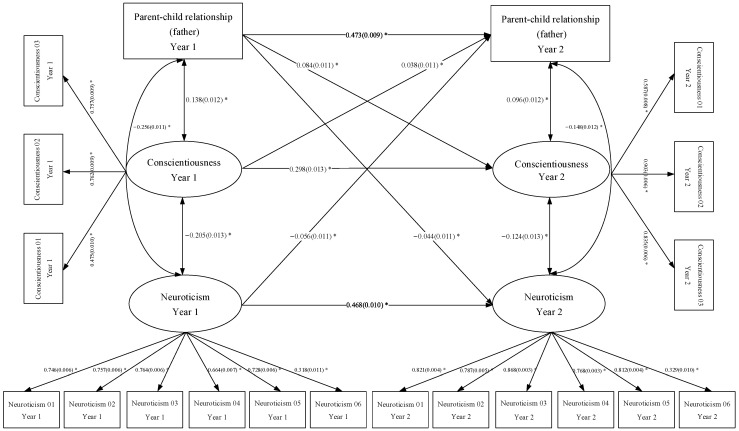
Bidirectional relationships between the parent‒child relationship (father), neuroticism and conscientiousness. Note: We use “*” to indicate a statistically significant relationship (*p* < 0.05) between variables.

Based on the characteristics of the variables, we constructed the cross-lagged models shown in Figure 1 and Figure 2. Figure 1 shows the bidirectional relationships between the parent‒child relationship (mother), neuroticism and conscientiousness. The model demonstrates good indices of fit: RMSEA = 0.048, 90 Percent C.I. = [0.046, 0.049]; CFI = 0.955, TLI = 0.946; SRMR = 0.040. In Figure 1, we can see that the standardized autoregressive path coefficient for the parent‒child relationship (mother) is 0.454 (*p* < 0.01); for conscientiousness, the standardized autoregressive path coefficient is 0.299 (*p* < 0.01); and for neuroticism, the standardized autoregressive path coefficient is 0.470 (*p* < 0.01). Figure 2 shows that the standardized autoregressive path coefficient for the parent‒child relationship (father) is 0.473 (*p* < 0.01); for conscientiousness, the standardized autoregressive path coefficient is 0.298 (*p* < 0.01); and for neuroticism, the standardized autoregressive path coefficient is 0.468 (*p* < 0.01). Controlling for autoregression, during the transition from seventh to eighth grade, Chinese middle school students’ parent‒child relationship (mother) significantly and positively predicts conscientiousness development one year later and significantly and negatively predicts neuroticism development one year later. In the meantime, Chinese middle school students’ conscientiousness significantly and positively predicts the parent‒child relationship (mother) one year later, whereas neuroticism significantly and negatively predicts the parent‒child relationship (mother) one year later. Similarly, the results of the bidirectional relationships between parent‒child relationship (father), neuroticism and conscientiousness demonstrated in Figure 2 are consistent with the conclusions from Figure 1. Thus, we confirm the proposed research Hypothesis 3.

## 5. Discussion

The descriptive statistical results demonstrate that the level of closeness of the parent‒child relationships decreases slightly from grade seven to grade eight, while conscientiousness also shows a decrease, and neuroticism shows an increasing trend. In particular, although the overall level of closeness in the parent‒child relationships remains moderate, the slight decline warrants attention. The finding is consistent with previously observed trends in personality traits and changes in parent–child relationships among adolescents from Germany, Spain, the United States and other countries [53,54,55]. This may be due to the transition from childhood to adolescence, which middle school students experience, where they begin to exhibit rebellious attitudes, develop a sense of independent self-identity, become more sensitive to external evaluations and relationships, and have less frequent communication with their parents, resulting in a decrease in the closeness of the parent‒child relationships [17,33,35]. In the meantime, the decline in conscientiousness may be due to the significant academic pressure imposed by the Chinese education system, as middle school students face the challenges of academic advancement and various exams [36,37]. They are also influenced by peers and societal expectations regarding a sense of responsibility, which may lead to a decrease in their diligence and commitment to tasks and obligations [11,16,17,18], resulting in a decline in proactive responsibility taking. Neuroticism, as a major component of personality, reflects an individual’s emotional stability and ability to regulate emotions. It is a predictor of various psychological and physical disorders and an indicator of an individual’s quality of life and life expectancy [19,20,21,22,23]. The increase in neuroticism reflects a negative trend in the psychological and emotional development of individuals [16]. This can be attributed to the physical and physiological changes, which middle school students undergo, along with the challenges of accelerated learning pace, social pressures and identity formation, making them more prone to emotional instability, feeling stressed, anxious and lacking in self-confidence. These phenomena need to be addressed and taken into account by parents and school teachers. In addition, we found a significant positive correlation between parent‒child relationships and adolescent conscientiousness, which is consistent with previous related literature [9,47,48]. The significant negative correlation between parent‒child relationships and neuroticism is consistent with previous research findings from countries such as the United Kingdom, Israel and the United States [28,44,45], indicating that adolescent mental health problems have indeed become a global concern.

Based on cross-lagged models, this study further validates the bidirectional predictive relationships between parent‒child relationships, neuroticism and conscientiousness, highlighting the important role of parent‒child relationships in adolescent development. Specifically, first, Chinese adolescents’ parent‒child relationships significantly positively predicted subsequent conscientiousness development, and conscientiousness levels also significantly positively predicted subsequent parent‒child relationships. These findings suggest that an intimate and harmonious parent‒child relationship provides adolescents with a supportive and nurturing family environment, which helps cultivate their conscientious traits. This supportive environment promotes adolescents’ diligence and commitment to tasks and obligations, helping them develop self-discipline and a sense of responsibility. At the same time, adolescents’ high levels of conscientiousness are an indicator of better parent‒child relationships, meaning that their behavior and actions may be more in line with parental expectations, thus promoting the maintenance of a healthy parent‒child relationship. Previous studies have predominantly examined the correlational relationship between parent‒child relationships and conscientiousness, focusing primarily on Western countries [9,16,18,48,49,50]. This study builds on the literature by further investigating this bidirectional relationship in the context of Chinese adolescents, providing new evidence on the bidirectional relationship between parent‒child relationships and conscientiousness development.

Second, Chinese adolescents’ parent‒child relationships significantly negatively predicted subsequent neuroticism development, and neuroticism levels also significantly negatively predicted subsequent parent‒child relationships. This finding is consistent with the conclusions of previous research by Hazel et al. (1982), Yu et al. (2019) and Brouillard et al. (2018) and supports the unidirectional negative impact of parent‒child relationships on adolescent neuroticism [9,25,46]. Our study also adds to the understanding of neuroticism as a predictor of parent‒child relationships. Positive parent‒child relationships provide emotional support and a sense of security, enabling adolescents to cope better with the stresses and challenges of life. This emotional support helps reduce anxiety and tension in adolescents, thereby reducing their levels of neuroticism. On the other hand, lower levels of neuroticism indicate a closer relationship between adolescents and their parents, as reduced emotional fluctuations and emotional stability contribute to establishing more stable and harmonious family communication. It is worth noting that previous studies have often combined the effects of fathers and mothers when analyzing parent‒child relationships in a general way. Our study comprehensively highlights the significant impact of parent‒child relationships on adolescent neuroticism and conscientiousness development by examining mother–child and father–child relationships separately.

In conclusion, our study not only supports existing theoretical perspectives but also provides insights for valuable educational practice. The ecological systems theory emphasizes the importance of the environment in the process of individual development and views individual development as the result of interactions between the individual and the surrounding environment. Among these environments, the family is the innermost microsystem within the ecological environment, closely related to the individual and exerting the greatest influence [29]. As a pivotal component of the family ecological environment, the parent‒child relationship plays an undeniably positive role in adolescent development and psychological well-being. According to the stress-buffering model, positive factors in one environment buffer the impact of risk factors in another environment on adolescent developmental outcomes [30]. The results of this study suggest that positive relationships and support from parents help prevent potential negative emotional effects and contribute to individuals becoming more responsible and healthier.

## 6. Implications for Educational Practice

The findings provide valuable insights for educational practice. Through empirical analysis of longitudinal data, we clarified the contributions of both mother–child and father–child relationships in promoting adolescent development in the Chinese educational context. This highlights the critical role of both mothers and fathers in ensuring the healthy development of children and emphasizes the need for each parent to be actively involved in fulfilling the family’s educational responsibilities. In China, fathers often occupy a marginalized or even absent position in family education, while mothers typically take on more responsibility for accompanying and caring for their children within the household [41]. Nevertheless, a burgeoning body of literature has begun to shed light on the positive effects of father–child relationships on adolescent emotions, behaviors and cognition [57,58,59]. Our study adds to this body of evidence by highlighting the indispensable role of both fathers and mothers in promoting conscientiousness and mitigating neuroticism among Chinese middle school students. This is in line with the family education law promulgated by the Chinese government last year, which emphasizes the paramount importance of joint parental involvement and participation in promoting the comprehensive development of minors. To begin with, both parents should recognize this importance and work together to improve parent–child bonding. They should give priority to encouraging parent–child interaction and providing emotional support to create a nurturing and supportive family atmosphere [60]. It is important to avoid stereotyping fathers and instead encourage their active involvement in intimate interactions and communication with their children. For instance, participation in democratic family meetings and volunteering in the community can be beneficial. In addition, parents should improve cooperation with schoolteachers, increase the frequency of communication and stay attuned to their child’s academic performance and emotional fluctuations. At the same time, appropriate support and motivation are essential to help children develop self-discipline, responsibility and effective time management skills. Finally, a child’s decreasing conscientiousness and increasing neuroticism may indicate problems in the parent–child relationship. This finding suggests that parents can introspect and make timely adjustments to address these concerns. Certainly, further research should include comparative studies across different cultural and educational backgrounds, exploring the mechanisms through which father–child and mother–child relationships affect adolescents’ personality and psychological well-being. An in-depth exploration of these aspects would be valuable if the relevant data could be obtained.

Based on a large-scale longitudinal survey, this study used a cross-lagged model to empirically clarify the bidirectional relationships between parent‒child relationships, neuroticism and conscientiousness among Chinese middle school students. The findings further enhance our understanding of the role, which parent‒child relationships play in shaping the development of neuroticism and conscientiousness among middle school students. Moreover, the study provides meaningful evidence within the context of the Chinese educational background.

## 7. Limitations

First, it should be noted that the information on parent‒child relationships, neuroticism and conscientiousness used in this study was obtained from student questionnaires. While students’ perceptions of parent‒child relationships may provide a more accurate reflection of the actual closeness between parents and children, self-report measures are inherently subject to certain biases. Additionally, due to the utilization of secondary data and the exceptionally large sample size, certain fit indices are not applicable. Future research could consider employing multiple methods and incorporating multiple sources of information (such as in-depth interviews with both students and their parents, as well as direct observation) to assess parent‒child relationships, thereby obtaining a more comprehensive and accurate understanding.

Second, due to the limited availability of data in the database, this study was based on two waves of survey data, and a two-wave cross-lagged model was constructed to explore the bidirectional relationships among Chinese adolescents’ parent‒child relationships, neuroticism and conscientiousness. If future releases of the CEPS project include additional waves of longitudinal data, further analysis could be conducted to explore the dynamic patterns of these variables over a longer time span.

Third, this study primarily focused on examining the predictive effects of parent‒child relationships on neuroticism and conscientiousness development. We also acknowledge that in real-life situations, the positive effects of parent‒child relationships extend beyond the variables addressed in this study. Researchers can explore the influence of parent‒child relationships on other aspects of middle school students’ performance. Future research can broaden the scope to include the school and community environments, analyzing the effects of relationships with teachers, neighbors or peers on the development of middle school students. Finally, it is necessary to clarify the matter of correlations in the context of a large sample size. Some of the relatively small correlations observed in this study may be due to the large sample size.

## 8. Conclusions

It is noteworthy that during the seventh and eighth grades among Chinese middle school students, the closeness of the parent‒child relationship with both parents decreased, accompanied by a decrease in conscientiousness levels and an increase in neuroticism levels. This highlights the need for parents and teachers to closely monitor and address the emotional and personality development of adolescents to prevent further deterioration in their mental health.

Second, among Chinese middle school students, there is a bidirectional predictive relationship between parent‒child relationships, conscientiousness and neuroticism development. Specifically, the levels of closeness in parent–child relationships show bidirectional positive predictive relationships with conscientiousness and bidirectional negative predictive relationships with neuroticism. These findings highlight the important role of close parent–child relationships in fostering later conscientiousness and mitigating neuroticism. Furthermore, decreasing conscientiousness and increasing neuroticism may serve as potential indicators for identifying problems within the parent–child relationship.

Third, both mothers and fathers play a crucial role in the healthy development of children, and each party should contribute to the family’s educational responsibilities. It is essential for both parents to support and cooperate with each other and to make joint efforts to create a warm and harmonious family environment and to foster high-quality parent‒child relationships. Such efforts are conducive to the development of children’s conscientiousness and their healthy growth.

## Figures and Tables

**Table 1:** Descriptive statistics of parent‒child relationships, neuroticism and conscientiousness.

Year	Variables	N	Mean	Standard Deviation	Min	Max	Skewness	Kurtosis
Year1	1. Parent‒child relationship (mother)	8437	2.747	0.483	1	3	−1.710	5.046
	2. Parent‒child relationship (father)	8437	2.629	0.553	1	3	−1.159	3.348
	3. Conscientiousness	8437	3.424	0.616	1	4	−1.507	5.835
	4. Neuroticism	8437	1.920	0.710	1	4.833	1.064	4.557
Year2	5. Parent‒child relationship (mother)	8437	2.707	0.498	1	3	−1.397	3.937
	6. Parent‒child relationship (father)	8437	2.506	0.579	1	3	−0.683	2.482
	7. Conscientiousness	8437	3.155	0.757	1	4	−0.92	3.560
	8. Neuroticism	8437	2.095	0.820	1	4.833	0.873	3.694

**Table 2:** Correlation analysis of parent‒child relationships, neuroticism and conscientiousness.

Year	Variables	1	2	3	4	5	6	7	8
Year1	1. Parent‒child relationship (mother)	1							
	2. Parent‒child relationship (father)	0.480 ***	1						
	3. Conscientiousness	0.105 ***	0.109 ***	1					
	4. Neuroticism	−0.241 ***	−0.249 ***	−0.169 ***	1				
Year2	5. Parent‒child relationship (mother)	0.471 ***	0.234 ***	0.087 ***	−0.164 ***	1			
	6. Parent‒child relationship (father)	0.240 ***	0.493 ***	0.086 ***	−0.184 ***	0.442 ***	1		
	7. Conscientiousness	0.100 ***	0.104 ***	0.241 ***	−0.129 ***	0.143 ***	0.134 ***	1	
	8. Neuroticism	−0.157 ***	−0.163 ***	−0.067 ***	0.441 ***	−0.216 ***	−0.221 ***	−0.156 ***	1

Note: *** *p* < 0.01.

## Data Availability

Data will be made available upon request.
